# The functional role of Nudt2 in human triple negative breast cancer

**DOI:** 10.3389/fonc.2024.1364663

**Published:** 2024-04-23

**Authors:** Rasha Abu-Rahmah, Hovav Nechushtan, Sanaa Hidmi, Amichay Meirovitz, Ehud Razin, Tamar Peretz

**Affiliations:** ^1^ Department of Oncology, Hadassah Hebrew University Medical Center, Jerusalem, Israel; ^2^ Department of Biochemistry and Molecular Biology, Institute for Medical Research Israel-Canada, the Hebrew University of Jerusalem, Jerusalem, Israel; ^3^ Department of Oncology, Soroka-Ben Gurion University Medical Center, Beer-Sheeva, Israel

**Keywords:** NUDT2, triple negative breast cancer (TNBC), Anchorage-independent growth, migration, invasion

## Abstract

The main known function of Nudix hydrolase 2 (Nudt2) is to hydrolyze the secondary messenger diadenosine 5’, 5’’’-p1, p4-tetraphosphate (Ap4A). In this study we examined the role of Nudt2 in breast carcinoma through its expression in human invasive ductal carcinoma tissues, and its functions in human triple negative breast cancer (TNBC) cell lines. A significantly higher expression of Nudt2 was observed in human invasive ductal carcinoma tissues compared to that in normal breast tissue. Knockdown of Nudt2 in TNBC cell lines resulted in a significant reduction in cellular proliferation via the Ki67 marker, accompanied by G0/G1 phase cell cycle arrest, in the migration and invasion of these cells and in tumorigenicity and anchorage-independent growth. It can therefore be concluded that Nudt2 plays a significant role in promoting TNBC growth.

## 1 Introduction

### 1.1 Overview of triple negative breast cancer (TNBC)

Female breast cancer is one of the most frequently diagnosed cancers and is a leading cause of cancer death (1). Among the different types of breast cancers, triple negative breast cancer (TNBC) is the most aggressive and has distinctive metastatic patterns with a poor prognosis (2). TNBC is characterized by the absence of expression of the estrogen receptor (ER), progesterone receptor (PR), and human epidermal growth factor receptor 2 (HER2) (2). TNBC is classified into basal-like 1 and 2, mesenchymal, and luminal androgen receptor subgroups (3, 4). Despite global research efforts, response to therapy following initial treatment of TNBC primary tumors with DNA-damaging agents and androgen receptors, varies considerably and is suboptimal (4). Therefore, elucidating the proteins and signaling pathway mechanisms involved in the metastatic process of this cancer has the potential to reveal new therapeutic targets. However, it is essential to proactively anticipate and explore novel resistance mechanisms that may arise with the development of these therapies. By employing robust models and diligent research, we can progressively discover the metastasis factors and recurrence (5).

### 1.2 Role of Nudt2 in breast cancer

Nudix hydrolase 2 (Nudt2) hydrolyzes diadenosine 5′, 5′′′‐p1, p4‐tetraphosphate (Ap4A) to yield AMP and ATP (6, 7). Nudt2 has been found to be associated with RAG proteins which are involved in mTOR activation in breast cancer cells (7). A recent study has revealed that Nudt2 was significantly higher in ductal breast carcinoma tissue compared to healthy tissue, highlighting this protein’s potential as a prognostic factor in human breast carcinomas (8). This study also showed Nudt2 to be an estrogen-repressed gene that is induced by HER2 pathways in breast carcinoma cells thereby promoting proliferation (8). We found in our recent study that Nudt2 knockdown suppressed anchorage-independent growth of human melanoma cells *in vitro*, and its effect *in vivo* was determined by investigating the role played by Nudt2 knockdown on the ability of the cells to form tumors in a mice xenograft model (9). Building upon these findings that Nudt2 promotes proliferation and might serve as a valuable and potential prognostic marker in human breast carcinomas, our main objective in the present study was to explore the role of Nudt2 in TNBC cells. We also wanted to determine its impact on the functional pathways of these cells. Significantly, our experiments revealed a substantial reduction in cell proliferation, tumorigenicity, migration and invasion following Nudt2 knockdown in TNBC cell lines. These findings underscore the pivotal role played by Nudt2 in these cellular processes and suggest its potential contribution to the aggressive nature of TNBC.

## 2 Material and methods

### 2.1 Patients and tissues

In this study, we employed two distinct sets of frozen tissue specimens. The initial set composed of matched breast IDC and normal breast tissues derived from the same TNBC patients (n=7). The second set consisted of breast IDC sourced from different TNBC patients and different normal breast tissues (normal tissues=2,tumor tissues=4).These tissue specimen were produced from our institution’s pathology laboratory and were subsequently preserved at -80°C within the Israeli National Tissue Bank for Research. All the patients provided informed consent before undergoing surgery and allowing examination of their tissue specimens.

### 2.2 Immunohistochemistry

Immunohistochemical IHC) staining was performed by Gavish Research Services on 7µm frozen sections using the Leica Bond max system (Leica Biosystems Newcastle Ltd, UK). Slides were dried at room temperature following pretreatment with epitope-retrieval solution (ER1, Leica Biosystems Newcastle Ltd, UK) followed by incubation with anti-Nudt2 ((#10484-1-AP, dilution 1:200, Proteintech, Biotest, Israel). Detection was performed using the Leica Bond Polymer Refine HRP kit (DS980 by Leica Biosystems Newcastle Ltd, UK). All slides were counter-stained with Hematoxylin and evaluated as a semi- quantitative analysis of the immunohistochemical reaction for NUDT2 was performed using a scoring scale as follows:

Grade 0= no positive reaction at all.
Grade 1= Only few cells are immune positive (<5 cells per a X20 field).
Grade 2= mild immune reaction (5-15 cells per a X20 field).
Grade 3= Moderate immune reaction (15-25 cells per a X20 field).
Grade 4= strong immune reaction (25-50 cells per a X20 field).
Grade 5= Marked immune reaction (>50 cells per a X20 field).

### 2.3 Cell culture

MDA-MB-231 and MDA-MB-436 human breast carcinoma cell lines, which are TNBC cells, were purchased from American Type Culture Collection (ATCC) (Manassas, USA). Cells were cultured in Dulbecco’s Modified Eagle Medium (DMEM) (#01-052-1A, Biological Industries, Israel) containing: 10% fetal bovine serum, 100 units/ml penicillin, 100 μg/ml streptomycin (#03-031-5C, Biological Industries, Israel) and 1 mM sodium pyruvate (#03-042-1B, Biological Industries, Israel).

### 2.4 Stable Sh-RNA lentiviral particle transfected triple negative breast cancer cell lines

Human breast carcinoma cell lines were cultured in 6-well plates (1.5x10^5^ cells/well) to reach 60% confluence on the first day of infection. They were infected with either Nudt2 shRNA lent viral particles (#sc-60188-V; Santa Cruz Biotechnology, Almog, Israel) or control shRNA lentiviral particles (# sc-108080; Santa Cruz Biotechnology) using Polybrene transfection reagent (#TR-1003-G; Merck, Israel) reaching a final concentration of 8 μg/ml. Cells were then incubated at 37°C, in a 5% CO_2_ incubator for 24 h, after which the medium was replaced with complete medium. After a further 24 h, the medium was replaced with puromycin (Cas-58-58-2, TOKU-E, Tiva-Biotech, Israel) selection medium for 48-96 h; the concentration was optimized for each cell line according to their killing curves.

### 2.5 Immunoblotting and immunoprecipitation (IP)

Human breast carcinoma cell lines and frozen human breast carcinoma tissues (whole cell extracts) were extracted on ice for 10mins by RIPA lysis buffer containing 50 mM Tris-HCl, 1% Nonidet P-40, 0.25% Na-deoxycholate, 150 mM NaCl, 1 mM EDTA, X1 protease inhibitor cocktail, 1 mM phenylmethylsulfonyl fluoride, 1 mM sodium orthovanadate, and 17 mM NaF. Proteins were resolved using 15% SDS-PAGE under reducing conditions and transferred to polyvinylidene difluoride membranes (Merck Millipore, Israel). Visualization of the proteins was performed by chemiluminescence with EZ-ECL (#20-500-1000, Biological Industries, Israel). For immunoprecipitation (IP), cells were washed twice with ice-cold 1XPBS and lysed with hypotonic lysis buffer (20 mM Tris-HCL pH=7.4, 10MmNacl, 3Mm Mgcl2), followed by 10 mins on rotation at 4°C. For cytoplasmic extraction, centrifugation at 14,000 rpm for 15 min at 4°C was performed. The resulting pellet was sonicated at 1 min intervals for 1 min on ice 3 times, followed by centrifugation at 14,000 rpm for 15 min at 4°C. Nucleus lysis buffer (20mM Hepes pH 7.9, 0.4M NaCl, 1mM EDTA, 1mM EGTA, 1mM DTT, protease inhibitor cocktail, PMSF, SOV and NaF) was then added. For IP, 2 μg of antibody was added to the nucleus lysate and incubated at 4°C overnight under gentle agitation with protein G magnetic beads (#1614023, Bio-Rad, Israel). The immunoprecipitate was washed 3 times with washing buffer (lysis buffer: 1X PBS, ratio (1:1)) and was subjected to SDS-PAGE and immunoblotting.

### 2.6 Cell proliferation

Cells were seeded at a density of 2500 cells per well in 96-well plates, with three wells per group. After 24 h, 48 h, 72 h and 96 h viability was detected by adding 50 µl of Cell Proliferation Kit (XTT based) solution (#20-300-1000, Biological Industrial) to each well. Absorbance was measured at an optical density of 450 nm using a plate reader (infinite M200 PRO).

### 2.7 Immunofluorescence staining

Cells were grown on glass slides at density of 250x10^3^/slide, and fixed with 4% paraformaldehyde for 20 min at room temperature, followed by permeabilization with 0.5% Triton X-100 in phosphate-buffered saline (1XPBS), cell blocking for an hour at room temperature in 0.5% BSA in 1XPBS, and incubation in primary antibody in blocking buffer overnight at 4°C. The primary antibodies utilized were: Ki67 monoclonal antibody (ab 16667), anti-beta II Tubulin [7B9] (ab28035) antibody; dilution was 1:100 for both antibodies. Appropriate secondary antibodies were applied for 1 h at room temperature. The secondary antibodies were: Cy2 –donkey anti-mouse IgG [H+L] and Cy3-goat anti-rabbit IgG [H+L, both were used at a dilution of 1:100. Samples were incubated with DAPI stain (Bio-Rad) at a dilution of 1:100 for 10–15 min. Coverslips were applied using Vectashield mounting medium for fluorescence (Vector-labs, Burlingame, CA USA) and slides were stored in the dark at 4°C until analysis was performed. Images were acquired using a ZEN 3.5 (ZEN lite) Axio Observer confocal Z1 laser scanning microscope, equipped with a 405, 488, and 561 laser. The images were analyzed by Qupath software.

### 2.8 Antibodies

Antibodies against Nudt2 (#10484-1-AP, dilution 1:1000, Proteintech, Biotest, Israel), β-actin (#A1978, dilution 1:10000, Sigma-Aldrich, Israel), Ki-67[SP6] (dilution 1:100, ab16667), anti-alpha tubulin antibody (dilution 1:100, ab7291); PUREBLU™ DAPI dye (#135-1303, dilution 1:100, Bio-Rad, USA), Cy™3 AffiniPure Goat Anti-Rabbit IgG (H+L) (# 111-165-144,dilution 1:100, Jackson, USA), Cy™2 AffiniPure Donkey Anti-Mouse IgG (H+L) (#715-225-151, dilution 1:100, Jackson, USA), Phospho-Rb (Ser807/811) Antibody (#9308,CST,USA), Phospho-Rb (Ser795) Antibody (#9301,CST,USA), Rb (4H1) Mouse mAb (#9309,CST,USA)and E2F-1 Antibody (#3742,CST,USA).

### 2.9 Cell cycle

MDA-MB-231 cells were seeded at 5x10^6^/well. After 96 h, the cells were collected and centrifuged at 2150 g for 5 min. The cells were then fixed with 1 ml 70% ethanol and incubated at 4°C for 15 min. At the end of incubation, the cells were washed with 1XPBS twice and centrifuged at 200 g for 5 min. The cells were then incubated with 500 PI solution (20 μg/ml PI, 0.0002g/ml RNase A, 0.01% Triton X-100) at 37°C for 30 min in the dark. After centrifugation at 200 g for 5 min, the cells were washed with 1 ml 1XPBS and then resuspended in 500 μl 1XPBS. Cell cycle distribution was calculated after appropriate gating of cell populations at an excitation wavelength of 488nm using CytoFLEX LX Flow Cytometer (Beckman Coulter). The data were analyzed using FCS Express 7 flow cytometry software.

### 2.10 Migration and invasion assay

The cell migration and invasion assay were performed by seeding 60x10^5^ of MDA-MB-231 and 90x10^5^ MDA-MB-436 cells/well in a 96-well. Image Lock plate in a CO_2_ incubator for 24 h to reach full confluence the next day. The scratch assay was performed using the 96-pin IncuCyte^®^ Wound Maker which creates precise and reproducible wounds in all wells of a 96-well Image Lock plate by gently removing the cells from the confluent monolayer. The plate was washed several times with DMEM medium, fresh medium was added to the wells, and the plate was placed in the IncuCyte^®^ Live-Cell Analysis System. RWD % was measured using IncuCyte software metrics every 2 h for a total of 48 h. For the invasion assay, the 96-well Image lock plate was coated with ECM matrigel 1µg/ml (E1270-1ml, Sigma, Israel) overnight, after 24h the cells were seeded following the removal of the matrigel and kept in a CO_2_ incubator until complete adherance around 3-4hours. A 96-pin IncuCyte^®^ Wound Maker was used to create wounds in all wells, the plate was then washed several times with DMEM medium and another layer of matrigel 0.5mg/ml was added. The plate was kept in the CO_2_ incubator for 30 mins for complete solidification, fresh MDEM medium was added and the plate was put in the IncuCyte^®^ Live-Cell Analysis System. RWD % was measured using IncuCyte software metrics every 2 h for a total of 48 h.

### 2.11 Soft agar colony formation assay

Soft agar colony formation assays were performed in 24-well plates. The lower layer was prepared from 0.8% noble agar, the top layer contained 1 x 10^4^ cells that were suspended in 0.3% noble agar in complete medium. After the agar solidified, 500 μl of complete medium were added. Cells were incubated at 37°C in a CO_2_ incubator for 21 days, fresh DMEM medium was added every 3-5 days after which the colonies were stained with p-iodonitrotetrazolium violet (#I8377, Sigma Aldrich, Israel). Images were captured using a Nikon SMZ25 stereomicroscope, and the number of cells were counted using Image J software (ij153-win-java8).

### 2.12 Statistical analysis

The Two Tailed Wilcoxon Signed-Rank Test, Mann-Whitney Test, Student’s t-test, Edgington’s Additive test and Fisher’s Chi-square tests were indicate in the statistic al analysis.

## 3 Results

### 3.1 Generation of stable Nudt2 knockdown TNBC cell lines

**Figure 1:**
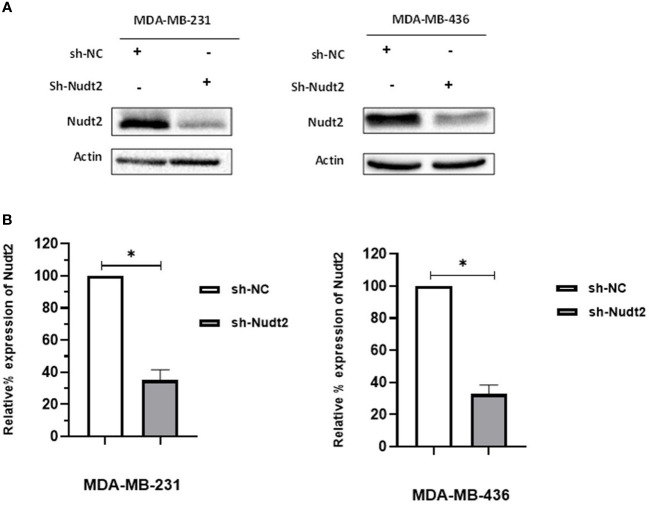
Generation of stable Nudt2 knockdown TNBC cell lines. MDA-MB-231 and MDA-MB-436 cell line were infected with shRNA against Nudt2 (sh-Nudt2) or non-targeting shRNA (sh-NC). **(A)** Western blot analysis of Nudt2 protein in both cell lines. One representative experiment is shown. **(B)** Relative expression level of Nudt2 normalized to β-actin expression. The relative expression was calculated by densitometric analysis of Western blots, with expression in control cells being 100% (n = 5). Results are represented as mean ± SEM. For statistical analysis, Wilcoxon signed-rank test was used (*p < 0.05).

We investigated whether Nudt2 plays a role in the behavior of TNBC cell lines by reducing Nudt2 levels in these cells. We generated control and Nudt2-knocked down human TNBC cells in two cell lines, MDA-MB-231 and MDA-MB-436, using either non-targeting or Nudt2-specific shRNA lentiviral particles. The stable knockdown of Nudt2 was verified in these cell lines via Western blot analysis (**Figure 1**) and mRNA expression level was verified using real-time PCR (**Supplementary Figure S1**).

### 3.2 Nudt2 expression in human breast invasive carcinoma tissues:

**Figure 2:**
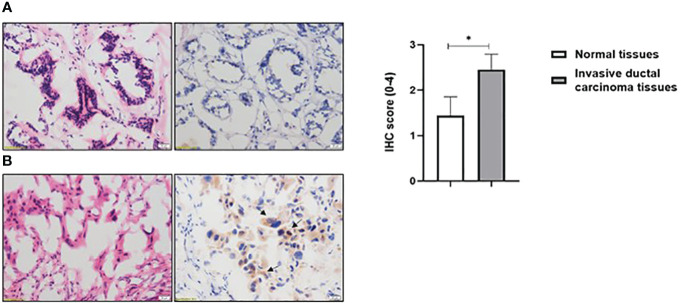
Immunohistochemical staining for Nudt2 in breast invasive ductal carcinoma (IDC) from TNBC patients. **(A)** Represent negative staining of Nudt2 in normal human breast tissue 20x with corresponding (H&E) 20X. **(B)** Represent strong positive staining of Nudt2 in human invasive ductal carcinoma (IDC) from TNBC patient 20x (black arrows) with corresponding (H&E). The data is presented as mean ± SEM, normal breast tissues n=9, breast invasive ductal carcinoma (IDC) tissues n=11. Edgington’s Additive test was used for statistical analysis yielding a *p* value of 0.0305.

**Figure 3:**
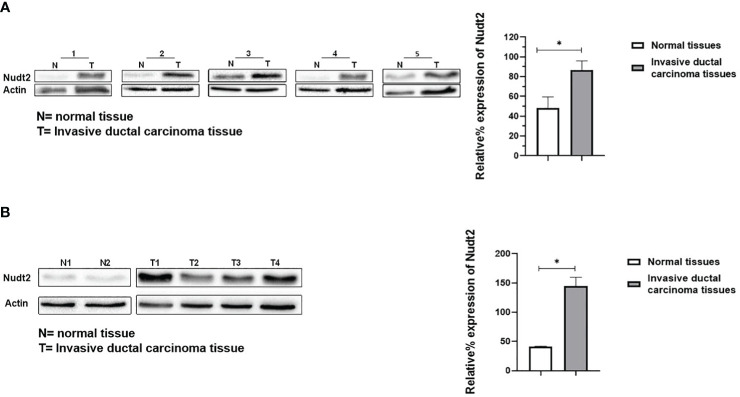
Expression of Nudt2 protein levels in human invasion ductal carcinoma (IDC). **(A)** Nudt2 expression in IDC tissues was higher than that in the normal breast tissues obtained from same TNBC patients. Expression was normalized to actin expression in the same sample. The data is presented as the mean ± SEM; n=7; *p*=0.039 by Student’s t-test. **(B)** Nudt2 expression in IDC tissues (n=4) sourced from different TNBC patients was higher than that seen in normal breast tissue (n=2). Mann-Whitney test was used for statistical analysis (*p*=0.0119) (*P<0.05). The probabilities were combined using Fisher’s Chi-square test yielding a p-values of 0.004035 (*P<0.05).

Nudt2 immunoreactivity was evaluated in two sets of human breast invasive ductal carcinoma (IDC) tissues. The first set consisted of seven samples of normal human breast tissue and of human IDC tissues sourced from the same TNBC patients. The second set comprised four tissues of human IDC tissues from different TNBC patients and two different human normal breast tissues. The results from immunohistochemistry (IHC) were scored, where a score of 1+ indicates weak staining,2+ mild staining, 3+ moderate staining and 4+ strong positive staining (refer to **Supplementary Table S1**). Our analysis revealed a significant elevation in Nudt2 levels in human IDC tissues compared to normal breast tissues (p < 0.05), as depicted in **Figure 2**. Similarly, immunoblotting analysis demonstrated a significantly higher Nudt2 protein level in human IDC tissues (p < 0.05) compared to normal breast tissues, as illustrated in **Figure 3**.

### 3.3 Associations between Nudt2 status and various clinic pathological parameters in IDC.

Based on the results of Nudt2 expression in the two sets of human breast invasive ductal carcinoma (IDC) tissues. We aimed to investigate the relationship between Nudt2 expression and clinicopathological parameters in IDC patients outlined in **Table 1**.

**Table 1:** Association between Nudt2% expression in tumor (%Ex Tumor) and in normal tissues (%Ex Normal) and various clinicalpathological parameters IDC patients.

No.	Age (Y)	T stage	N stage	Grade	%Ex Tumor	%Ex - Normal
1	66	2	1	3	70	16.5
2	62	1	0	2	115	54
3	38	2	1	3	102	78
4	65	2	0	2	99	20.5
5	70	1	0	3	41	99
6	48	1	0	3	78	33
7	27	2	0	3	100	42
8	83	2	0	3	158	NA
9	55	2	0	2	170	NA
10	30	2	0	2	150	NA
11	68	2	0	3	100	NA

Nudt2 expression levels correlated with (TNM) system which refers to the international standard for classifying the malignant of a tumor based on factors: tumor (T), node (N) and metastases (M). All patients in the study were categorized as no metastases at diagnosis (M0), tumor (T) stage 1-2, nodal involvement (N) stages 0-1 and Grades 2-3.Our findings revealed that Nudt2 expression showed higher expression in 90% of the IDC cases that assessed in both sets.

### 3.4 Effects of Nudt2 expression on cell proliferation in TNBC cells:

**Figure 4:**
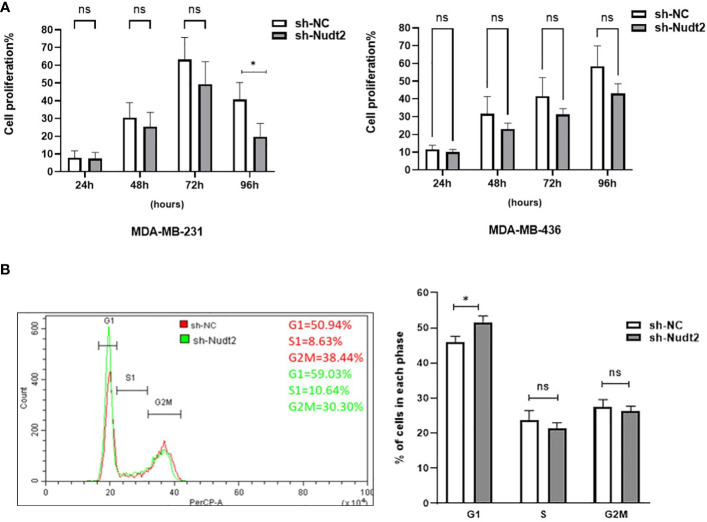
The role played by Nudt2 in the proliferation of the TNBC MDA-MB-231 cell line. **(A)** Cell viability percentages of MDA-MB-231 and MDA-MB-436 cell lines at 4 time points (24h, 48h, 72 and 96h). No significant reduction was observed at 24h (*p=0.99*),48h (*p=0.91*) and 72h (*p=0.79*), *p=0.91*, *p=0.79*, ns *p>0.05*. Following 96 h incubation, a significant reduction in proliferation observed in the sh-Nudt2 (Nudt2 knockdown) of MDA-MB-231 cell line (*p=0.026*) (*P<0.05). In contrast, there was no significant reduction in proliferation at 24h, 48h, 72, 96h was observed in the sh-Nudt2 of the MDA-MB-436 cell line (*p>0.99, p>0.99, p=0.87* and *p=0.40* respectively, ns *p>0.05*), for statistical analysis, Mann-Whitney Test was used. **(B)** MDA-MB-231 cells were stained with propidium iodide (PI). DNA content was measured by flow cytometry. Percentages of cells in the G1, S and G2M phases were determined using FCS express cell cycle analysis. One experiment was representative, the data are presented as individual experiments with the mean ± SEM (MDA-MB-231, n=7) showing the significant increasing of G1 phase at 96h in sh-Nudt2 compared to sh-NC, *P>0.05* as calculated by the Mann-Whitney Test.

The XTT assay was used out to assess the viability and proliferation of TNBC MDA-MB-231 and MDA-MB-436 cell lines at 24, 48, 72 and 96 h. The proliferation of Nudt2 knockdown MDA-MB-231 cells was significantly reduced at 96 h compared to that of wild-type cells *(p>0.05)*. Conversely, there was no significant reduction in proliferation observed in MDA-MB-436 cells at any of the time points (**Figure 4A**).

Additionally, we corroborated these results by validating cell proliferation in both cell lines through colony formation assay,as depicted in **Supplementary Figure S3**. Subsequent cell cycle analysis demonstrated that the G1 phase was significantly increased leading to cell arrest in knockdown MDA-MB-231 cells at 96h time point, however no significant alteration was found in S1 and G2M phases (**Figure 4B**). These results indicate that Nudt2 knockdown reduces the proliferation of MDA-MB-231 cells and plays a significant role in the regulation of cellular proliferation.

### 3.5 Expression of the Ki67 marker in the proliferation of the TNBC MDA-MB-231 cell line:

**Figure 5:**
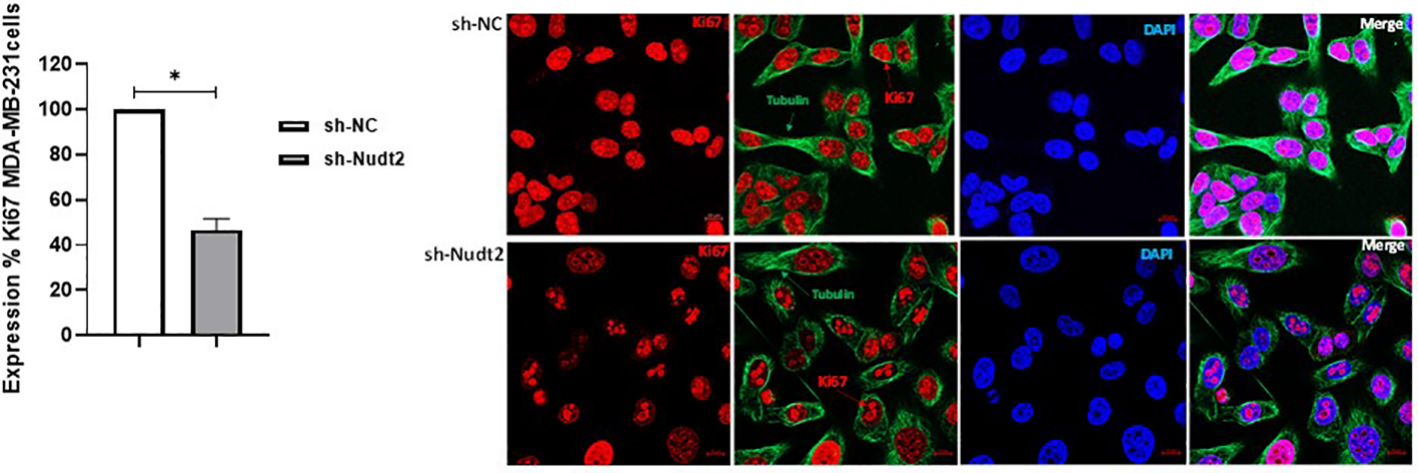
Cell proliferation as detected through Ki-67 expression in the TNBC MDA-MB-231 cell line. Ki-67 expression was significantly reduced in Nudt2 knockdown cells. Images were acquired using a ZEN 3.5 (ZEN lite) Axio Observer confocal Z1 laser scanning microscope equipped with a laser of 405, 488, and 561 nm. Ki-67 expression was determined by normalizing to the sh-NC cells. Red- Ki-67, blue- nuclear DNA (DAPI), and green- tubulin. The images were analyzed by Qupath software (MDA-MB-231, n=3). This image represents one of several images acquired during the experiments. The data are presented as mean ± SEM. For statistical analysis, Wilcoxon signed-rank test was used, *p < 0.05.

Ki-67 protein levels and localization vary throughout the cell cycle (10). Since this marker’s expression is associated with phases of cell cycle during mitosis, it is often assumed that Ki-67 is required for cell proliferation and that its down regulation might promote cell cycle arrest. Our results showed that the expression of Ki-67 protein in Nudt2 knockdown MDA-MB-231 cells was 50% less than that of the control MDA-MB-231 cells at 96h time point (**Figure 5**).

### 3.6 Nudt2 knockdown suppresses human TNBC MDA-MB-231 cell proliferation through G0/G1 cell-cycle arrest:

**Figure 6:**
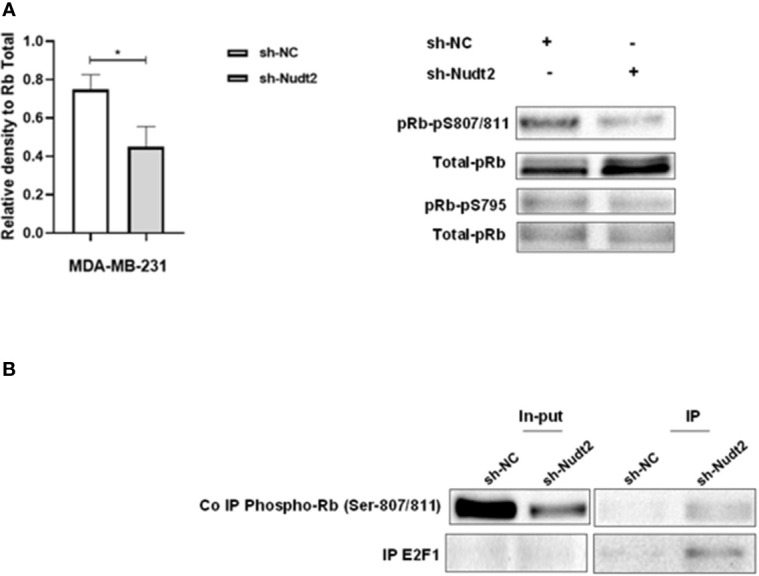
Expression of phosphorylated Rb in the TNBC MDA-MB-231 cell line. **(A)** Western blotting detection of phosphorylated Rb sites 795 and 807/811; and the ratio of signal intensity of pRb phospho-S807/811 and S 795 to total Rb levels in control and Nudt2 knockdown MDA-MB-231 cells. A significant reduction in phosphor-S807/811 was detected in the Nudt2 knockdown cells compared to the wild type cells. The results represent one of five independent experiments with similar results. The Wilcoxon-test was used for statistical analysis (*p*=0.03), *P>0.05. **(B)** Immunoprecipitation (IP) showed higher pRb phospho807/811-E2F1 association in the Nudt2 knockdown MDA-MB-231 cell line compared to wild type cells.

We observed a significant increase in the G1 phase of the Nudt2 knockdown MDA-MB-231 cell line compared to wild type cells at 96h time point (**Figure 3B**). Western blot analysis of the expression and the phosphorylation of the G0/G1 cell cycle regulatory protein retinoblastoma (Rb) in the nucleus, was performed (11). Significant hypophosphorylation of Rb at residue S807/811 was observed in the Nudt2 knockdown MDA-MB-231 cell line. Additionally, higher pRb phospho S807/811-E2F1 association was observed in Nudt2 knockdown MDA-MB-231 cell line at 96h time point (**Figure 6**). We also verified the absence of Rb expression in the MDA-MB-436 cell line (**Supplementary Figure S4**).

### 3.7 The role played by Nudt2 in cell migration and invasion in TNBC cell lines:

**Figure 7:**
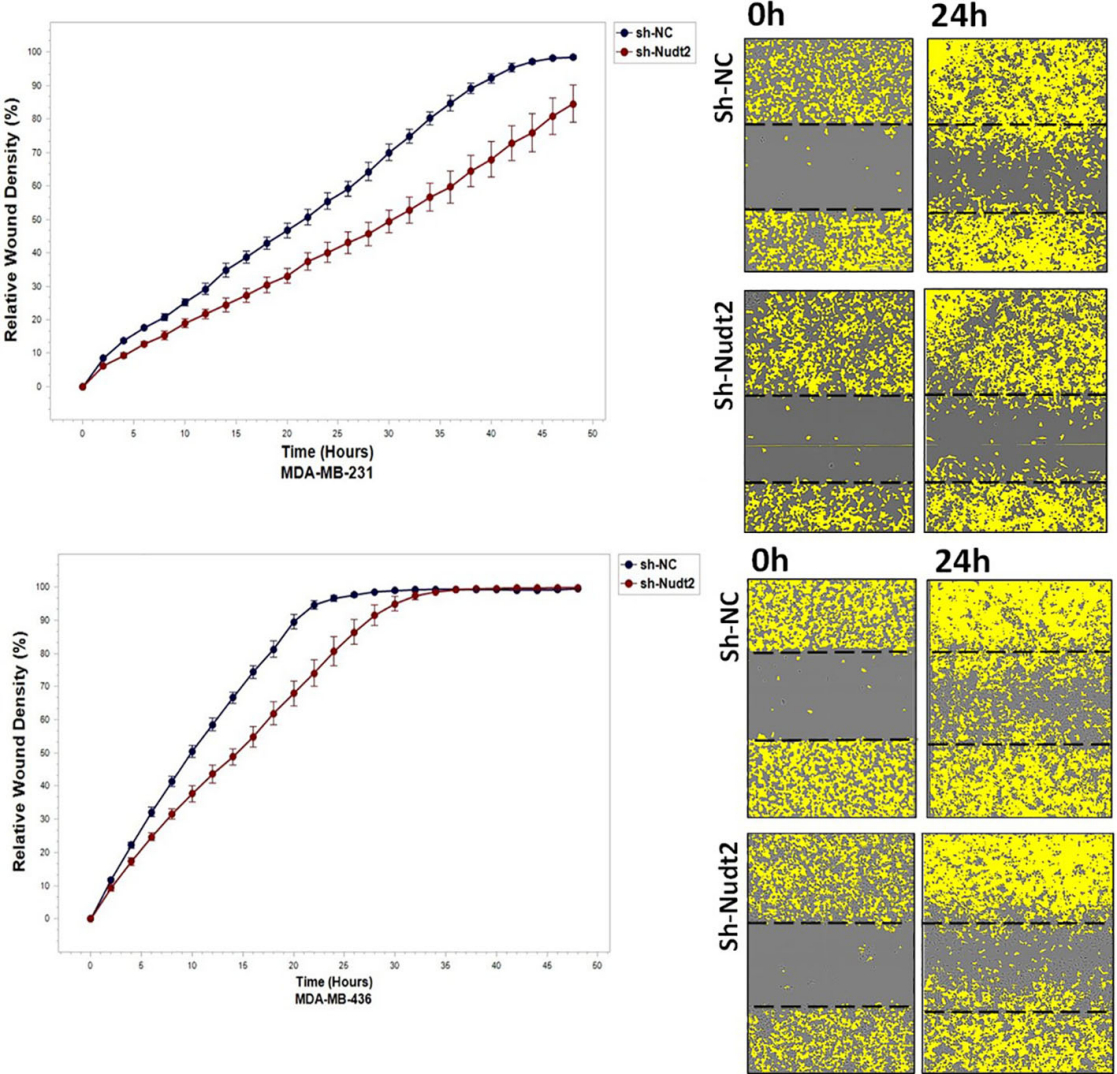
The role effect of Nudt2 knockdown on the migration of TNBC MDA-MB-231 and MDA-MB-436 cell lines. Nudt2 knockdown resulted in a significant reduction in migration in both cell lines using the scratch wound assay with the IncuCyte live imaging system. Relative wound density (RWD %) was measured using IncuCyte software metrics every 2 h over a period of 48 h. Area under the curve (AUC) was computed by numerical integration. Data are presented as individual experiments (MDA-MB-231 n=5; MDA-MB-436 n=5), *p* values were calculated for the comparison between sh-NC AUC and sh-Nudt2 AUC, *p>0.005..

**Figure 8:**
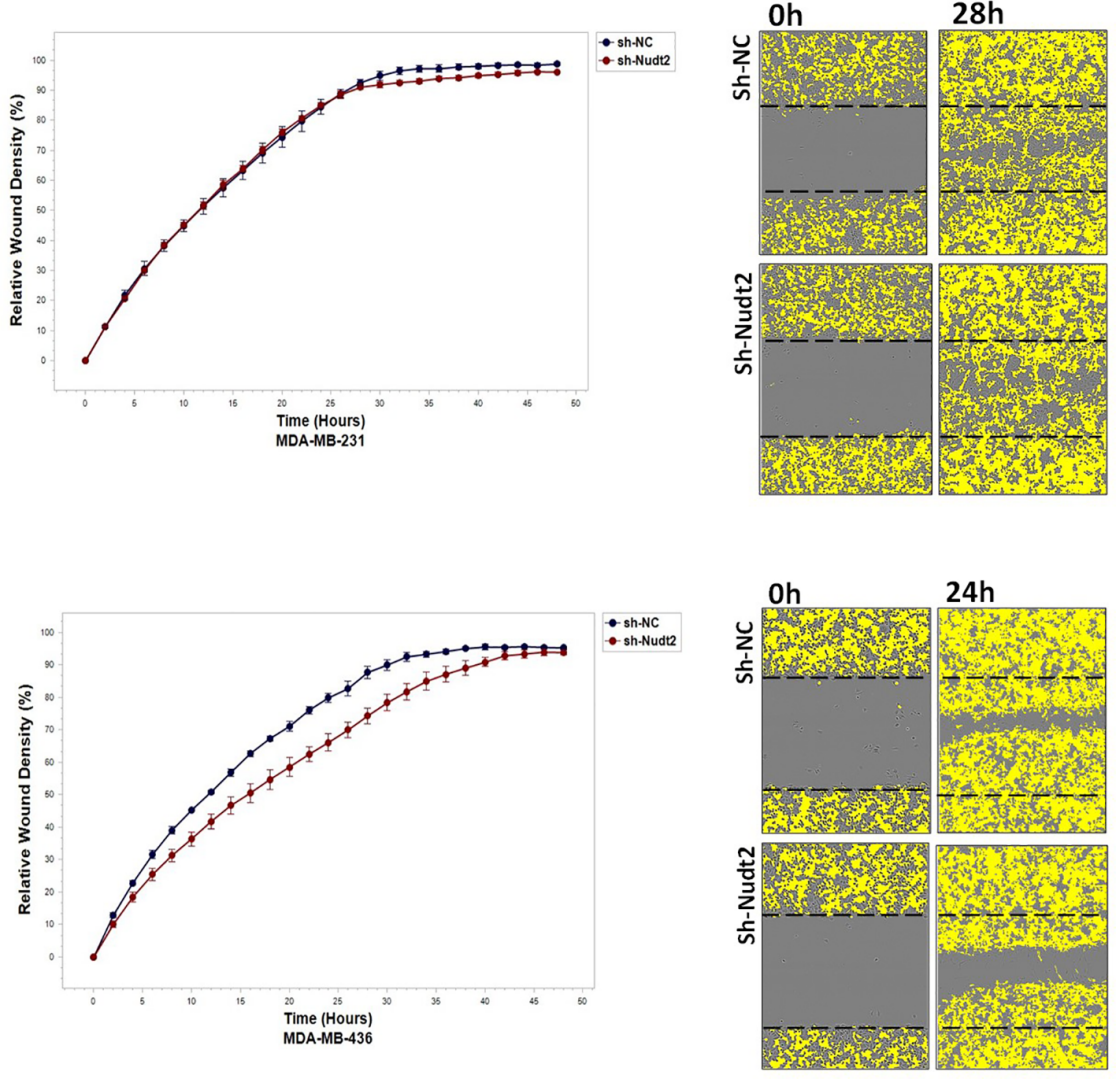
The effect of Nudt2 knockdown on invasion in TNBC MDA-MB-231 and MDA-MB-436 cell lines. Nudt2 knockdown resulted in a significant reduction in invasion in both cell lines using IncuCyte live imaging system. In MDA -MB-231 cells, the reduction started at 28 h. Relative wound density (RWD %) was measured by using IncuCyte software metrics every 2 h over period of 48 h. Area under the curve (AUC) was computed by numerical integration. Data are presented as individual experiments (MDA-MB-231 n=5; MDA-MB-436 n=5), *p* values were calculated for the comparison between sh-NC AUC and sh-Nudt2 AUC, *p>0.005.

To study the migration of the cancer cells, the wound scratch assay was performed in TNBC MDA-MB-231 and MDA-MB-436 cells. Cell migration and invasion were determined as a percentage of relative wound density (RWD) every 2 h for 48 h. The results revealed that the migration and the healing speed in both sh-Nudt2 TNBC cell lines were significantly slower than that of the sh-NC cells. Nudt2 knockdown resulted in a median area under the curve (AUC) that was significantly decreased (*p>0.005)* in both MDA-MB-231 and MDA-MB-436 cell lines. In the invasion assay, the healing rate of sh-Nudt2 MDA-MB-436 cells were significantly slower than that of control cells from the start of the assay. However, in MDA-MB-231 cells the healing rate was significantly slower only after 28 h. Nudt2 knockdown resulted in a decreased AUC (RWD% vs. hours), with a significant reduction (*p>0.005)* seen in both MDA-MB-231 and MDA-MB-436 cell lines (**Figures 7**, **8**). These results clearly demonstrated that Nudt2 promoted migration and invasion in breast cancer cells.

### 3.8 Nudt2 expression in tumorigenicity of TNBC cell lines:

**Figure 9:**
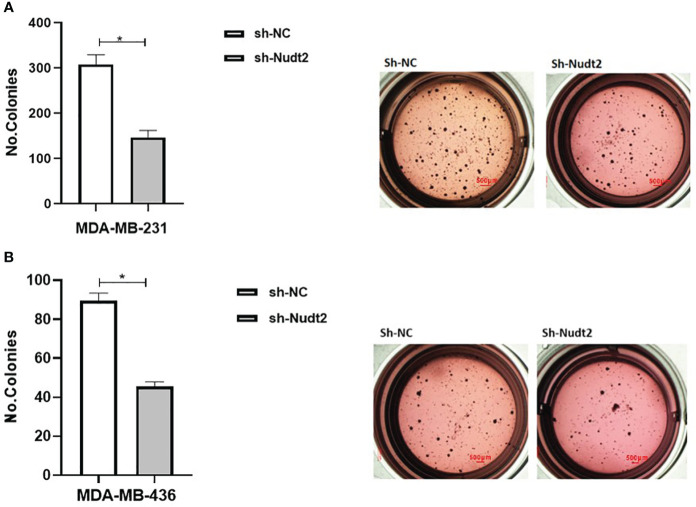
Effect of Nudt2 knockdown on anchorage-independent growth in MDA-MB-231 and MDA-MB-436 cell lines. Soft agar colony formation experiments were performed by seeding 10000 cells/well in 24-well plates for 21 days. Colonies were stained with 1 mg/ml of p-iodonitrotetrazoliumviolet stain and were counted using Image J software. There was a significant reduction in the number of colonies in MDA-MB-231 sh-Nudt2 cells **(A)** and in MDA-MB-436 sh-Nudt2 cells **(B)** compared to control cells. Data are presented mean ± SE, n=5, *p>0.05 calculated by Wilcoxon Test.

Anchorage-independent growth is a fundamental parameter in cancer biology as it has been linked to tumor cell aggressiveness *in vivo*, by way of tumorigenic and metastatic properties, and has also been utilized as a marker for *in vitro* transformation. Soft agar colony formation is typically used for a broad range of applications documenting the tumorigenicity of cancer cells. Soft agar colony formation was performed with MDA-MB-231 and MDA-MB-436 cell lines to assess the behavior of cancer cells with reduced Nudt2 expression. As can be seen in **Figure 9**, colony numbers and sizes in sh-Nudt2 MDA-MB-231 and MDA-MB-436 cells were significantly less than those of control cells (*p>0.05).*

## 4 Discussion

In our study we observed a significant elevation in the expression levels of Nudt2 in two distinct sets of human IDC tissues (**Figures 2**, **3**). These tissue sets were exclusively sourced from patients diagnosed with TNBC. This finding suggests an important role of Nudt2 in human TNBC and aligns with what was published previously (8). These studies highlight the idea thatNudt2 may serve as a valuable prognostic marker in breast cancer. It was recently discovered that Nudt2 has a significant role in promoting breast cancer proliferation by different mechanisms involving estrogen (7, 8). Previously it was reported that Nudt2 is involved in breast cancer proliferation by regulating mTORC1 localization (7). Nudt2 could also promote the proliferation of breast carcinoma cells through a decrease in the intracellular Ap4A level. Previous studies demonstrated that Ap4A is involved in the cell cycle by slowing the process of replication, which subsequently allows cells to repair possible DNA damage in ovarian cell lines (12). Our study found a significant reduction in cell proliferation associated with a significant increase in the G0/G1 phase in the Nudt2 knockdown MDA-MB-231 cell line (**Figure 4**). The Ki-67 protein, which plays a role in mitotic cells, has been widely used as a proliferation marker for human tumor cells (10). We found reduced expression of Ki67 markers in the Nudt2 knockdown MDA-MB-231 cells (**Figure 5**), which confirmed our results of cell cycle arrest in the G0-G1 phase in human TNBC MDA-MB-231 cells. Rb is an important cell cycle regulatory marker (13). Rb proteins repress gene transcription, which is required for transition from the G1 to S phase, by directly binding to the transactivation domain of E2F1 (13). In G0 and early G1, hypo-phosphorylated Rb physically associates with E2F1 factors and blocks their transactivation domain. In late G1, hyper-phosphorylated Rb releases E2F1 factor, allowing for the expression of genes that encode products necessary for S-phase progression. In this present study we found that hypo-phosphorylated Rb in the Nudt2 knockdown MDA-MB231 cell line had higher Rb-E2F1 association (**Figure 6**).

This delay in the release and transition of the E2F1 transcriptional factor from late G1 to S phase leads to G0/G1 arrest. No significant reduction in the proliferation of MDA-MB-436 cells was seen. This could be attributed to the fact that these cells are Rb null cells, meaning they do not express the Rb protein (14).

We also explored the impact of Nudt2 knockdown on migration and invasion. Our results clearly showed a significant reduction in both migration and invasion in both human Nudt2 knockdown MDA-MB-231 and MDA-MB-436 cell lines (**Figures 7**, **8**). Further investigation is required to unravel the underlying molecular mechanism responsible for Nudt2’s influence on migration and invasion.

This study demonstrated that Nudt2 knockdown inhibited anchorage-independent growth in two human TNBC cell lines: MDA-MB-231 and MDA-MB (**Figure 9**). Anchorage-independent growth is a hallmark of cancer and is necessary for metastasis (15). Anchorage-independent growth is also connected to epithelial-to-mesenchyme transition (EMT) in many cancers (16).

## 5 Conclusion

In our present study, higher expression levels of Nudt2 were detected in human IDC tissues, linking it to the promotion of proliferation of breast carcinoma cells. Additionally, our results showed that Nudt2 is important in the maintenance of anchorage independent growth and the reduction of cell migration and invasion. The underlying mechanisms of Nudt2’s role in migration and invasion should be investigated in future studies. The limitation of the current study is that it is based on *in vitro* models only, future work will aim to corroborate the results in an *in vivo* model.

## Data availability statement

The original contributions presented in the study are included in the article/**Supplementary Material**. Further inquiries can be directed to the corresponding author.

## Ethics statement

The studies involving humans were approved by the committee on research involving human subjects of the Hebrew University-Hadassah Medical School, The committee on research involving Human Subjects reviewed the research application 0346-12-HMO- Of- Prof Tamar Peretz. The studies were conducted in accordance with the local legislation and institutional requirements. The participants provided their written informed consent to participate in this study.

## Author contributions

RA: Validation, Visualization, Investigation, Methodology, Software, Writing – original draft. ER: Validation, Visualization, Funding acquisition, Resources, Supervision, Writing – review & editing. HN: Supervision, Writing – review & editing. SH: Conceptualization, Investigation, Methodology, Supervision, Writing – review & editing. AM: Supervision, Writing – review & editing. TP: Investigation, Supervision, Validation, Writing – review & editing.
