# Supplementary material for: The functional role of Nudt2 in human triple negative breast cancer
Source: Front Oncol. 2024 Apr 23;14:1364663. doi: 10.3389/fonc.2024.1364663 (PMC11075069; doi:10.3389/fonc.2024.1364663)
Supplement: Supplementary file 1 [file DataSheet_1.zip › Helsinki forms/genetic permission .pdf]

Hadassah
University
Hospital

THE COMMITTEE ON RESEARCH INVOLVING HUMAN SUBJECTS

Of

THE HEBREW UNIVERSITY-HADASSAH MEDICAL SCHOOL

Jerusalem, Israel

The committee on research involving Human Subjects reviewed the research application 0346-12-HMO- Of- Prof Tamar Peretz

on "Characerization of genetic material, including total genome/exome sequencing. From tumor cells
as a predictive and prognostic measure, and as a tool to follow up treatment in cancer patients".

After reviewing the report on the progress of the research, continuation is hereby authorized until 31/03/2023.

Prof. Eran Leitersdorf, M.D.
Chairman of the Helsinki Committee
Hadassah Medical Organization
