# Supplementary material for: The functional role of Nudt2 in human triple negative breast cancer
Source: Front Oncol. 2024 Apr 23;14:1364663. doi: 10.3389/fonc.2024.1364663 (PMC11075069; doi:10.3389/fonc.2024.1364663)
Supplement: Supplementary file 1 [file DataSheet_1.zip › Helsinki forms/PARP1217_012433314.pdf]

DARP 1217

שם פרטי:	ג'ורג'
שם משפחה:	סוסקי
מס' תעודת זהות:	012433314
תאריך:	18.1.2016
חתימה:	

פרטי וחתימת מקבל ההסכמה מדעת:
ההסכמה הנ"ל התקבלה על ידי, לאחר שהסברתי למשתתף/ת במחקר את האמור לעיל ווידאתי

שהסבריי הובן על ידו/ה.

שם פרטי:	לוי
שם משפחה:	לוס
תפקיד:	אחות
תאריך:	18.1.16
חתימה וחותמת:	

<u>הצהרת החוקר הראשי</u>

אני מתחייב לקיים את כל הוראות הדין הקשורות במחקרים רפואיים בבני-אדם ולהקפיד על כל הסייגים האתיים ובכלל זאת, העקרונות המופיעים בהצהרת הלסינקי ובשבועת הרופא.

חתימה:	תאריך:
