# Supplementary material for: The functional role of Nudt2 in human triple negative breast cancer
Source: Front Oncol. 2024 Apr 23;14:1364663. doi: 10.3389/fonc.2024.1364663 (PMC11075069; doi:10.3389/fonc.2024.1364663)
Supplement: Supplementary file 1 [file DataSheet_1.zip › Helsinki forms/PARP1284_055414833.pdf]

PARP 1284

שם פרטי:	
שם משפחה:	
מס' תעודת זהות:	
תאריך:	
חתימה:	

פרטי וחתימת מקבל ההסכמה מדעת:

ההסכמה הנ"ל התקבלה על ידי, לאחר שהסברתי למשתתף/ת במחקר את האמור לעיל ווידאתי

שהסברי הובן על ידו/ה.

שם פרטי:	
שם משפחה:	
תפקיד:	
תאריך:	
חתימה וחותמת:	

<u>הצהרת החוקר הראשי</u>

אני מתחייב לקיים את כל הוראות הדין הקשורות במחקרים רפואיים בבני-אדם ולהקפיד על כל הסייגים האתיים ובכלל זאת, העקרונות המופיעים בהצהרת הלסינקי ובשבועת הרופא.

חתימה:	תאריך:
