# Supplementary material for: The functional role of Nudt2 in human triple negative breast cancer
Source: Front Oncol. 2024 Apr 23;14:1364663. doi: 10.3389/fonc.2024.1364663 (PMC11075069; doi:10.3389/fonc.2024.1364663)
Supplement: Supplementary file 1 [file DataSheet_1.zip › Helsinki forms/PARP1445_054373287.pdf]

BARP 1445

**שם פרטי:**	[illegible]
**שם משפחה:**	[illegible]
**מס' תעודת זהות:**	5437 3287
**תאריך:**	8.8.2016
**חתימה:**	[illegible]

פרטי וחתימת מקבל ההסכמה מדעת:

ההסכמה הנ"ל התקבלה על ידי, לאחר שהסברתי למשתתף/ת במחקר את האמור לעיל ווידאתי

שהסברי הובן על ידו/ה.

**שם פרטי:**	[illegible]
**שם משפחה:**	[illegible]
**תפקיד:**	[illegible]
**תאריך:**	8/8/16
**חתימה וחותמת:**	[illegible]

<u>הצהרת החוקר הראשי</u>

אני מתחייב לקיים את כל הוראות הדין הקשורות במחקרים רפואיים בבני-אדם ולהקפיד על כל הסייגים האתיים ובכלל זאת, העקרונות המופיעים בהצהרת הלסינקי ובשבועת הרופא.

**חתימה:**	**תאריך:**
