# Supplementary material for: The functional role of Nudt2 in human triple negative breast cancer
Source: Front Oncol. 2024 Apr 23;14:1364663. doi: 10.3389/fonc.2024.1364663 (PMC11075069; doi:10.3389/fonc.2024.1364663)
Supplement: Supplementary file 1 [file DataSheet_1.zip › Helsinki forms/PARP1491_031503899.pdf]

PARP 1491

שם פרטי:	[illegible]
שם משפחה:	מזרחי
מס' תעודת זהות:	031503899
תאריך:	12/9/2016
חתימה:	[signature]

פרטי וחתימת מקבל ההסכמה מדעת:

ההסכמה הנ"ל התקבלה על ידי, לאחר שהסברתי למשתתף/ת במחקר את האמור לעיל ווידאתי

שהסברי הובן על ידו/ה.

שם פרטי:	[illegible]
שם משפחה:	[illegible]
תפקיד:	[illegible]
תאריך:	12/9/16
חתימה וחותמת:	[signature]

<u>הצהרת החוקר הראשי</u>

אני מתחייב לקיים את כל הוראות הדין הקשורות במחקרים רפואיים בבני-אדם ולהקפיד על כל הסייגים האתיים ובכלל זאת, העקרונות המופיעים בהצהרת הלסינקי ובשבועת הרופא.

חתימה:	תאריך:
