# Supplementary material for: The functional role of Nudt2 in human triple negative breast cancer
Source: Front Oncol. 2024 Apr 23;14:1364663. doi: 10.3389/fonc.2024.1364663 (PMC11075069; doi:10.3389/fonc.2024.1364663)
Supplement: Supplementary file 1 [file DataSheet_1.zip › Helsinki forms/PARP277_051885275.pdf]

PARP 272

טופס הסכמה מדעת להשתתפות בניסויים גנטיים – הסכמה להשתתף בניסוי הכולל איסוף, אחסון או בדיקת DNA. לקוח מתוך נוהל משרד הבריאות לניסויים גנטיים (נספח 23 פרק 30)

לטופס הסכמה מדעת יצורף דף הסבר למשתתפים אשר יכיל את המידע הנדרש למשתתף על מנת להחליט אם ברצונו להשתתף במחקר. על דף ההסבר להיות כתוב בשפה וברמה המובנת למשתתף ולכלול בין היתר מידע על ההיבטים המייחדים סוג מחקר זה ממחקרים אחרים.
הועדה דורשת כי בטופס ההסכמה מדעת תיכלל הצהרה על הפוטנציאל המסחרי, כולל פוטנציאל לפטנטים. בדף ההסבר יש להתייחס לתוצאות המסחריות העשויות לנבוע משימוש בתוצאות המחקר והזכויות – אם קיימות כאלו – של המשתתפים.

0-5188527-5 ע.כ.
אינגלס
ורדה
ש.ל: 1953
שרות: 388 יח: 138800
30/07/2012
מ.ע:

אני החתום מטה:

**שם פרטי**	ורדה	**שם משפחה**	אינגלס
**מס' תעודת זהות**	5188527-5		
**כתובת**	הנרקיס 64 ירושלים	**מיקוד**	96629
**מטרות הניסוי**			

מטרת החוקרים היא לבדוק גנים שעשויים להיות מעורבים ב: הופעת סרטן (מחלה או מצב) וגנים אשר עשויים להשפיע על רמת הסיכון לחלות בסרטן (מחלה או מצב). בכוונת החוקרים להשתמש בדגימת ה-DNA שלי עבור המטרות הבאות:
הערכת תרומת המוטציות בגנים BRCA1 ו – BRCA2 לתחלואה בסרטן השד, שחלה, פרוסטטה, מלנומה, מעי ולבלב. בדיקת האינטראקציה שבין המוטציות ובין גורמים סביבתיים והורמונליים. מידע זה מהוה בסיס להמלצות למעקב וטיפול. ראה פירוט בדף מידע לחולה המצורף.

שם ונושא הניסוי (תיאור קצר): בדיקת מדגם מייצג של חולי סרטן שד, שחלה, פרוסטטה, מלנומה, מעי ולבלב לנוכחות מוטציות בגנים BRCA1 ו – BRCA2.
אני מצהיר בזה כי אני מסכים/ה להשתתף בניסוי הכולל איסוף, אחסון, ו/או בדיקת DNA כמפורט במסמך זה.

אני מצהיר בזה כי הוסבר לי על ידי (שם הרופא / חוקר / המסביר):
כי החוקר הראשי (שם הרופא/ה) ד"ר כדורי קיבל ממנהל המוסד הרפואי אישור לביצוע הניסוי בבני אדם, כמשמעותו בתקנות בריאות העם (ניסויים רפואיים בבני אדם) התשמ"א – 1980 (להלן הניסוי).
כי הניסוי מתבצע בחסות מחלקה אונקולוגית.
כי לחוקר הראשי ולחוקרי המשנה יש __ או אין X (לסמן אחד) זיקה ליוזם הניסוי. (אם יש, פרט).
החוקרים הם יוזמי המחקר.

1. הסכמה וזכויות המשתתפים: מטרת טופס ההסכמה הינה לשאול האם אני מסכים לתרום דגימת דם (מלא מקור הדגימה ממנו יופק DNA עבור בדיקה גנטית לניסוי. אני רשאי לברר בכל עת את זכויותיי כמשתתף בניסוי. השתתפותי בניסוי נעשית בהתנדבות ועל פי הסכמתי ולא אקבל כל תמורה ישירה מהשתתפותי. אני חופשי/ה לבחור שלא להשתתף בניסוי, ואני חופשי/ה להפסיק בכל עת את השתתפותי בניסוי, מבלי לפגוע בזכויותיי. בכל בעיה הקשורה לניסוי, אוכל לפנות אל ד"ר לונה כדורי, ד"ר איילה הוברט, ד"ר אבירם ניסן (שם הרופא/ה) במספר טלפון 6777825.

2. הליך וטיפול בדגימות (לשנות במקרים של לקיחת דגימות אחרות). הוסבר לי כי כל אדם מיומן ייקח 5 סמ"ק (כמות) מדמי (לדוגמא, בערך 2 כפיות). (אם מקור ה-DNA אינו מנוזל הגוף או רקמתו – יש לציין) צוות הניסוי ישאל אותי על מצבי הרפואי, על טיפולים רפואיים, על ההיסטוריה הרפואית שלי, על ההיסטוריה הרפואית של משפחתי, ועל המוצא המשפחתי שלי. מידע זה הינו מידע רפואי אישי, ועל צוות הניסוי מוטלת החובה לשמור על סודיותו. החוקים ישתמשו בדגימת ה-DNA שלי למטרות שצוינו בטופס זה בלבד. אם החוקרים יבקשו להשתמש בדגימות ה-DNA שלי למרות נוספות, יהיה עליהם לקבל את הסכמתי לכך. הרופא / חוקר האחראי על הניסוי הסביר לי שימושים אפשריים נוספים לדגימה בעתיד.

א. אני מסכים שדגימת DNA שלי תשמש לכל ניסוי בעתיד שקיבל אישור כחוק.

חתימה: ____________________

ב. אני מסכים שדגימת DNA שלי תשמש לכל ניסוי בעתיד שקיבל אישור כחוק בנושא: גנטיקה של סרטן בלבד.

חתימה: ____________________

ג. אני מסכים שדגימת דם שלי תשמש רק לניסוי הנוכחי

חתימה: ____________________

ד. בתום הניסוי אני מסכים לשמור את דגימת ה-DNA שלי כדגימת DNA מזוהה ואת תוצאות הבדיקה כבדיקה גנטית מזוהה.

חתימה: ____________________

ה. אני מסכים כי מדגימות הדם שלי יכינו החוקרים שורות תאים תמידיות, באופן זה יוכלו החוקרים להמשיך להשתמש בדגימות הדם שלי ככל שיצטרכו לכל ניסוי שאושר כחוק.

חתימה: ____________________

הוסבר לי כי דגימת ה-DNA שנלקחה ממני תאוחסן ב: מעבדה לגנטיקה של האדם, בי"ח הדסה ע"כ (מקום). החוקרים ישמרו על הדגימות המזוהות למשך (   ) חודשים לאחר קבלת תוצאות הניסוי או למשך (10 שנים לאחר האיסוף). לאחר מכן ינהגו החוקרים בדגימה כפי שהסכמתי בסעיף (2) לטופס הסכמה הזה, ובהתאם לחוק ולתקנות של משרד הבריאות.
החוקרים יאחסנו את דגימת הדם שלי או הדגימה הגנטית שהוכנה מהדגימה שנתתי מדמי בחדר מוגן עם גישה מוגבלת. אך ורק החוקרים או אנשים העובדים עם החוקרים בניסוי זה, יוכלו לעבוד עם הדגימה שנלקחה ממני.
אם אני פורש/ת מהניסוי בשלב כלשהו, החוקרים עשויים לשמור את המידע שנאסף עלי עד השלב בו פרשתי מהניסוי, או שפי שצינתי בטופס זה.

ARP277

טופס הסכמה מדעת להשתתפות בניסויים גנטיים – הסכמה להשתתף בניסוי הכולל איסוף, אחסון או בדיקת DNA. לקוח מתוך נוהל משרד הבריאות לניסויים גנטיים (נספח 23 פרק 30)

3. סודיות המידע הגנטי: מידע אישי ותוצאות אשר מתקבלות ממשתתפי הניסוי, הינו מידע שקיימת לגביו זכות לפרטיות, ויוגן בהתאם להוראות כל דין. החוקרים יגבילו את הגישה למקומות בהם נשמר המידע הרפואי ותוצאות הבדיקות הגנטיות שנערכו לי. התיק הרפואי שלי כמטופל בכל מוסד רפואי לא יכיל כל תוצאות של ניסוי גנטי זה. מובטח כי זהותי האישית תשמר בסוד על ידי כל העוסקים והמעורבים בניסוי ולא תפורסם בכל פרסום כולל פרסומים מדעיים אלא אם נתתי את הסכמתי המפורשת לכך מראש ובכתב. בעלי תפקידים בניסוי עשויים לעיין במידע הרפואי עלי ובממצאים הגנטיים על מנת לבדוק אם הניסוי התבצע באופן נכון וכחוק. אנשים אלו כוללים את החוקרים, אנשים העובדים עם החוקרים בניסוי גנטי זה, יזם הניסוי, וועדת הלסינקי המוסדית ומפקחי משרד הבריאות. חובת הסודיות חלה גם על כל אחד מאלה. עם סיום הניסוי ולפי הכללים שיקבע משרד הבריאות, יופרדו הפרטים המזהים מהדגימות שנלקחו ממני ומתוצאות הבדיקה, אלא אם נתתי את הסכמתי לשמירה מזוהה של הבדיקה בסעיף (2) לעיל. ידוע לי כי אם אינני מסכים לשמור את דגימת ה-DNA שנלקחה ממני כדגימה מזוהה, ואם יופרדו מתוצאות הניסוי כל הפרטים המאפשרים לזהותי, לא ניתן יהיה להשתמש בהם בשום דרך בעתיד לטובתי ולטובת בני משפחתי.

הצהרת הרופא/ה:

ההסכמה הנ"ל נתקבלה על ידי וזאת לאחר שהסברתי למשתתף/ת בניסוי כל האמור לעיל וכן ווידאתי שכל הסברי הובנו על ידו / על ידה.

שם הרופא המסביר: ד"ר נעמה הלפרן

חתימה הרופא/ה וחותמת: ד"ר נעמה הלפרן מ.ר. 90272

תאריך: 14/8/12
