# Supplementary material for: The functional role of Nudt2 in human triple negative breast cancer
Source: Front Oncol. 2024 Apr 23;14:1364663. doi: 10.3389/fonc.2024.1364663 (PMC11075069; doi:10.3389/fonc.2024.1364663)
Supplement: Supplementary file 1 [file DataSheet_1.zip › Helsinki forms/PARP467_029008992.pdf]

RARS 487

שם פרטי:	0-2900899-2 ע.כ.
שם משפחה	פקטר
מס' תעודת	נעמי
תאריך:	ש.ל: 1972 ר.א: מחלקה: אונקולוגיה 05/10/2016 18:04
חתימה:	פידי: מיול עכ מ.ע:

05/10/2016 FHYAFFIT

החולה לא יכולה לחתום
בעלה מאשר

פרטי וחתימת מקבל ההסכמה מדעת:
ההסכמה הנ"ל התקבלה על ידי, לאחר שהסברתי למשתתף/ת במחקר את האמור לעיל ווידאתי

שהסברי הובן על ידו/ה.

שם פרטי:	
שם משפחה:	
תפקיד:	
תאריך:	9/10/16
חתימה וחותמת:	

ד"ר אלברט גרינשפון מ.ר. 108750 אונקולוגיה

<u>הצהרת החוקר הראשי</u>

אני מתחייב לקיים את כל הוראות הדין הקשורות במחקרים רפואיים בבני-אדם ולהקפיד על כל הסייגים האתיים ובכלל זאת, העקרונות המופיעים בהצהרת הלסינקי ובשבועת הרופא.

חתימה:	תאריך:

7. הסכמה להשתתפות במחקר

בחתימתך הנך מאשר/ת כי קראת את טופס ההסכמה מדעת והנך מוכן/ה להשתתף בניסוי זה, לאחר שהבנת את פרטיו ומשמעותו.

פרטי המשתתף/ת וחתימתו/ה:

**שם פרטי:**	[illegible]
**שם משפחה:**	[illegible]
**מס' תעודת זהות:**	
**תאריך:**	[illegible]
**חתימה:**	[illegible]

פרטי וחתימת מקבל ההסכמה מדעת:

ההסכמה הנ"ל התקבלה על ידי, לאחר שהסברתי למשתתף/ת במחקר את האמור לעיל ווידאתי שהסבריי הובן על ידו/ה.

**שם פרטי:**	[illegible]
**שם משפחה:**	
**תפקיד:**	[illegible]
**תאריך:**	[illegible]
**חתימה וחותמת:**	[illegible]

הצהרת החוקר הראשי

אני מתחייב לקיים את כל הוראות הדין הקשורות במחקרים רפואיים בבני-אדם ולהקפיד על כל הסייגים האתיים ובכלל זאת, העקרונות המופיעים בהצהרת הלסינקי ובשבועת הרופא.

**חתימה:**	**תאריך:**
