# Supplementary material for: The functional role of Nudt2 in human triple negative breast cancer
Source: Front Oncol. 2024 Apr 23;14:1364663. doi: 10.3389/fonc.2024.1364663 (PMC11075069; doi:10.3389/fonc.2024.1364663)
Supplement: Supplementary file 1 [file DataSheet_1.zip › Helsinki forms/PARP71_080238520.pdf]

PARP-71.

לטופס הסכמה מדעת יצורף דף הסבר למשתתפים אשר יכיל את המידע הנדרש למשתתף על מנת להחליט אם ברצונו להשתתף במחקר. על דף ההסבר להיות כתוב בשפה וברמה המובנת למשתתף ולכלול בין היתר מידע על ההיבטים המייחדים סוג מחקר זה ממחקרים אחרים.

הועדה דורשת כי בטופס ההסכמה מדעת תיכלל הצהרה על הפוטנציאל המסחרי, כולל פוטנציאל לפטנטים. בדף ההסבר יש להתייחס לתוצאות המסחריות העשויות לנבוע משימוש בתוצאות המחקר והזכויות – אם קיימות כאלו – של המשתתפים.

אני החתום מטה:

שם פרטי	שם משפחה:
מספר תעודת זהות:	
כתובת	מיקוד:
מטרות הניסוי:	

0-8023852-0 ע.כ.
מנסיאן
נ'ינה
ש.ל: 1943
מרפ אונקולוגית
19/04/2010
מ.ע:

מטרת החוקרים היא לבדוק גנים שעשויים להיות מעורבים ב: הופעת סרטן (מחלה או מצב) וגנים אשר עשויים להשפיע על רמת הסיכון לחלות בסרטן (מחלה או מצב).
בכוונת החוקרים להשתמש בדגימת ה- DNA שלי עבור המטרות הבאות:
הערכת תרומת המוטציות בגנים BRCA1 ו- BRCA2 לתחלואה בסרטן השד, שחלה, פרוסטטה, מלנומה, מעי ולבלב. בדיקת האינטראקציה שבין המוטציות ובין גורמים סביבתיים והורמונלים. מידע זה מהווה בסיס להמלצות למעקב וטיפול. ראה פירוט בדף מידע לחולה המצורף.

שם ונושא הניסוי (תיאור קצר): בדיקת מדגם מייצג של חולי סרטן שד, שחלה, פרוסטטה, מלנומה, מעי ולבלב לנוכחות מוטציות בגנים BRCA1 וBRCA-2.
אני מצהיר/ה בזה כי אני מסכים/ה להשתתף בניסוי הכולל איסוף, אחסון, ו/או בדיקת DNA כמפורט במסמך זה.

[illegible]ס הסכמה מדעת להשתתפות בניסויים גנטיים - הסכמה להשתתף בניסוי הכולל איסוף, אחסון או בדיקת DNA

לקוח מתוך נוהל משרד הבריאות לניסויים גנטיים (נספח 23 פרק 30)

א. אני מסכים שדגימת DNA שלי תשמש לכל ניסוי בעתיד שקיבל אישור כחוק.

חתימה: ______________

ב. אני מסכים שדגימת DNA שלי תשמש לכל ניסוי בעתיד שקיבל אישור כחוק בנושא: גנטיקה של סרטן בלבד.

חתימה: ______________

ג. אני מסכים שדגימת DNA שלי תשמש רק לניסוי הנוכחי.

חתימה: ______________

ד. בתום הניסוי אני מסכים לשמור את דגימת ה- DNA שלי כדגימת - DNA מזוהה ואת תוצאות הבדיקה כבדיקה גנטית מזוהה.

חתימה: ______________

ה. אני מסכים כי מדגימות הדם שלי יכינו החוקרים שורות תאים תמידיות. באופן זה יוכלו החוקרים להמשיך להשתמש בדגימות הדם שלי ככל שיצטרכו לכל ניסוי שאושר כחוק.

חתימה: ______________

[illegible]פס הסכמה מדעת להשתתפות בניסויים גנטיים - הסכמה להשתתף
בניסוי הכולל איסוף, אחסון או בדיקת DNA
[illegible]לקוח מתוך נוהל משרד הבריאות לניסויים גנטיים (נספח 23 פרק 30)

[illegible]צהרת הרופא/ה:

ההסכמה הנ"ל נתקבלה על ידי וזאת לאחר שהסברתי למשתתף/ת בניסוי כל האמור לעיל וכן וידאתי שכל הסברי הובנו על ידו/על ידה.

שם הרופא/ה המסביר:

חתימת הרופא/ה וחותמת: ______________________

תאריך: 19.4.2010
