# Supplementary material for: The functional role of Nudt2 in human triple negative breast cancer
Source: Front Oncol. 2024 Apr 23;14:1364663. doi: 10.3389/fonc.2024.1364663 (PMC11075069; doi:10.3389/fonc.2024.1364663)
Supplement: Supplementary file 2 [file DataSheet_2.pdf]

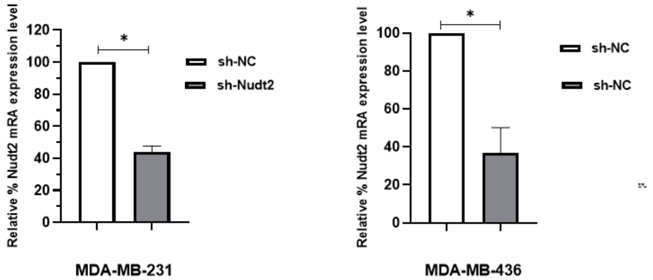

Figure S1.The Nudt2 knockdown in the triple negative breast cancer cell lines: MDA-MB-231 and MDA-MB-436 cells was confirmed by qPCR. Relative mRNA expression level was determined via qPCR (n = 4).). Results are represented as mean ± SEM. For statistical analysis, Wilcoxon signed-rank test was used. ($p < 0.05$)

Sample No.	Normal	Tumor
**1**	1	4
**2**	1	3
**3**	0	2
**4**	1	3
**5**	3	4
**6**	3	3
**7**	1	0
**8**	**3**	2
**9**	**1**	**NA**
**10**	**NA**	2
**11**	**NA**	2
**12**	**NA**	0

Table S1. The table presents the IHC scores for Nudt2 in the two sets of human breast invasive ductal carcinoma (IDC) tissues. samples both: the normal breast tissues and the IDC tissues

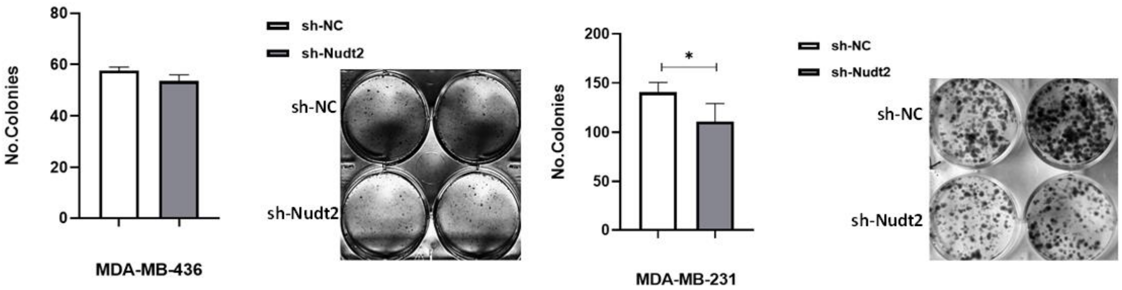

Figure S3. Colony formation assay was conducted to validate cell proliferation in the triple negative breast cancer cell lines: MDA-MB-231 and MDA-MB-436 cells. The finding indicates significant reduction in cell proliferation in MDA-MB-231 (n=6) (*p< 0.05),* in the contrast cell proliferation was found to be insignificant in MDA-MB-436 cells. Results are represented as mean ± SEM. For statistical analysis, Wilcoxon signed-rank test was used. This assay provides additional evidence regarding the impact of Nudt2 knockdown on cell proliferation in specific cell lines.

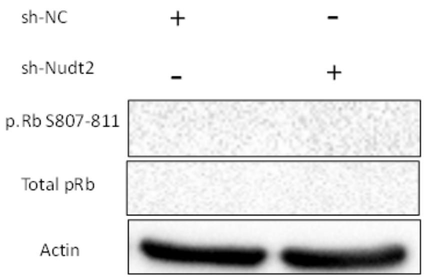

Figure S4. Immunoblotting result demonstrating the negative expression of the total Rb(retinoblastoma) protein and the phosphor Rb (S807/811) in the MDA-MB-436 cell line.
